# Improving the Quality of Reclaimed Water via Applying *Spirulina platensis* to Eliminate Residual Nitrate

**DOI:** 10.3390/ijerph20032117

**Published:** 2023-01-24

**Authors:** Xiaohua Jiang, Xin Shan, Fengmin Li

**Affiliations:** 1 College of Environmental Science and Engineering, Ocean University of China, Qingdao 266100, China; 2 Sanya Oceanographic Institution, Ocean University of China, Sanya 572000, China

**Keywords:** *Spirulina platensis*, nitrate removal, reclaimed water, nutrient deficiency, microalgae cultivation

## Abstract

The application of reclaimed water has been recognized as the key approach for alleviating water scarcity, while its low quality, such as high nitrogen content, still makes people worry about the corresponding ecological risk. Herein, we investigated the feasibility of removing residual nitrate from reclaimed water by applying *Spirulina platensis*. It is found that 15 mg/L total nitrogen could be decreased to 1.8 mg/L in 5 days, equaling 88.1 % removal efficiency under the optimized conditions. The deficient phosphorus at 0.5–1.0 mg/L was rapidly eliminated but was already sufficient to support nitrate removal by *S. platensis*. The produced ammonia is generally below 0.2 mg/L, which is much lower than the standard limit of 5 mg/L. In such a nutrient deficiency condition, *S. platensis* could maintain biomass growth well via photosynthesis. The variation of pigments, including chlorophyll a and carotenoids, suggested a certain degree of influences of illumination intensity and phosphorus starvation on microalgae. The background cations Cu^2+^ and Zn^2+^ exhibited significant inhibition on biomass growth and nitrate removal; thus, more attention needs to be paid to the further application of microalgae in reclaimed water. Our results demonstrated that cultivation of *S. platensis* should be a very promising solution to improve the quality of reclaimed water by efficiently removing nitrate and producing biomass.

## 1. Introduction

Water scarcity has emerged as a serious crisis globally due to the augmenting demand driven by population growth, economic development, and consumption upgrade [1]. It is recently reported that more than four billion people in the world are suffering from water scarcity in varying degrees of severity [2]. Increasing water supply capacity is a crucial approach towards alleviating water scarcity. Supplementary water sources can be obtained from unconventional means, such as desalination of seawater and reclamation of wastewater, depending on the local availability [3,4]. Municipal wastewater is currently recognized as a very promising alternative source to meet water demand after effective treatments [5]. However, inadequate water quality is still preventing the wide acceptance of treated wastewater for further reuse [6,7].

Reclaiming high-quality water from wastewater is definitely an important way to help solve the problem of water scarcity. There are some successful cases internationally, including the direct potable reuse in Windhoek, Namibia, and the groundwater replenishment in California, United States [8,9]. Moreover, Israel has recycled 60% of urban wastewater for agricultural irrigation, and Singapore has reused 5% of total water for industrial production [10,11]. In China, the treatment rate of municipal wastewater is increasing rapidly these years associated with the urbanization process, while the application of reclaimed water is very limited to a narrow range, such as toilet flushing and landscape greening [12,13]. This is likely due to concerns about the risk of reclaimed water, such as the substantial nitrogen compounds that may cause eutrophication of receiving waters [14,15].

In fact, many countries and regions have stipulated the concentration limit of total nitrogen (TN) level for sewage treatment and recycling, such as 5–10 mg/L TN for the United States and European Union and 15 mg/L for China [16,17]. However, the current concentration limits still cannot reduce the occurrence of corresponding ecological risks. It is reported that the emitted nitrogen from wastewater is mainly nitrate, resulting in the increase of dissolved inorganic nitrogen in the aquatic environment [18,19]. Accompanied by the intensification of global warming, water blooms and green tides are presumably to occur continuously if the nitrogen emissions cannot be effectively controlled [20].

It has to be admitted that removing nitrogen from wastewater effluent or reclaimed water remains a challenge because the conventional denitrification process with activated sludge commonly consumes a great deal of energy and chemicals [21]. The anammox technique has then become the research focus in the last decades, though the effective operation of the anammox system requires warm and ammonia-rich environmental conditions [22]. In recent years, some projects have tried to add an advanced treatment process to further remove TN, but the high operation cost and low removal efficiency are still real problems that we have to face [21]. Thus, a query is raised considering whether there exists a stable and low-cost approach for removing nitrogen from reclaimed water with a low carbon source and high nitrate proportion.

Microalgae culture could be a great option for this purpose by employing nitrogen nutrients as fertilizer during photosynthesis [23]. In other words, microalgae as the primary producer could assimilate inorganic nitrogen ions into biomass, so as to avoid the tedious cycle of nitrification, denitrification, and then nitrogen fixation [24]. Among the various species, *Spirulina platensis* is considered as the preferred option based on the following assumptions: the protein-rich organism should be hungry for nitrogen nutrients, the filamentous form seems likely to be beneficial to algae-water separation due to the agglomeration characteristics, and the harvested biomass is expected to be valuable in the field of commercial applications such as nutrition and feed additives [25,26]. It is reported that the application of *S. platensis* is effective in removing high concentrations of ammonia from wastewater [27]. However, it is still unclear whether *S. platensis* can survive in the reclaimed water and be employed to remove the low concentration of nitrate without organic carbon source and ammonia.

Hence, this work aims to study the feasibility of nitrate removal from reclaimed water by making use of *S. platensis*. According to the national standard of China, the initial nitrate level was set at 15 mg/L by considering the upper limit of tertiary effluent [17]. The critical parameters, such as initial inoculation amount, illumination intensity, and initial phosphorus level, were regulated to optimize the efficiency of nitrate removal. The biological adaptation of *S. platensis* was investigated by determining the variations of biomass growth and photosynthetic pigments. The effects of some background ions possibly present in reclaimed water were introduced into the culture solution to study their effects on the removal efficiency. It is anticipated from the current research to pave the way for further improving the quality of reclaimed water.

## 2. Materials and Methods

### 2.1. Chemicals and Materials

The microalgae strain (*S. platensis* FACHB-314 in Figure S1) was obtained from the Institute of Hydrobiology, Chinese Academy of Sciences (Wuhan, China). The algae cells were cultivated in Zarrouk medium as shown in Table S1, which was autoclaved before inoculation of algae cells. The cultivation of *S. platensis* was carried out as shown in Figure S2 in the artificial climate conditions (25 °C and 12/12 h light/dark cycle illumination at 2000 lux). All chemicals were of at least analytical grade unless specified and were used directly without any purification.

### 2.2. Batch Test

The cultured *S. platensis* was taken from the Zarrouk medium and centrifuged at 8000 rpm for 10 min to discard supernatant. The obtained biomass was washed three times with deionized water and re-suspended in 250 mL Erlenmeyer flasks containing 100 mL synthetic reclaimed water with 15 mg/L nitrate as displayed in Table S1. The composition of synthetic reclaimed water was a modification of the Zarrouk medium by mainly controlling the nutrients such as nitrate and phosphorus at very low conditions. Moreover, other compositions, including zinc and copper, are all within the lowest limit of Grade IA national discharge standard of pollutants for municipal effluent [17], which is the main source of reclaimed water in China. The cultivation of *S. platensis* was carried out under the same artificial climate conditions as described above except changing the initial inoculation amount, illumination intensity, and initial phosphorus level. Aliquots were then withdrawn periodically and analyzed immediately.

### 2.3. Analytical Methods

The dry cell weight (DCW, mg/L) of *S. platensis* was determined by the established relationship between DCW and OD_560_ (optical density at 560 nm) as follows:

DCW = 675.6 × OD_560_ + 32.0 (R^2^ = 0.997), (1)

The OD_560_ was analyzed by using a Metash UV-9000S UV-Vis spectrophotometer. The DCW value was calculated as the weight gain of a 0.45 μm filter paper by filtering 10 mL algal suspension after drying it at 105 °C for 2 h [28].

The contents of chlorophyll a and carotenoids were determined on the basis of the previously reported method [29]. In brief, samples were centrifuged at 10000 rpm for 10 min and washed with water three times. After discarding the supernatant, the obtained residues were mixed with methanol and incubated at 4 °C in the dark for 24 h. The further post-centrifuged supernatant could be finally analyzed spectrophotometrically at wavelengths of 665, 652, and 470 nm and computationally using the following equations:

[Chlorophyll-a] = 16.72 × A_665_ − 9.16 × A_652_, (2)

[Carotenoid] = (1000× A_470_ − 1.63 ×[Chlorophyll-a])/221, (3)

The water quality was measured by analyzing the levels of total nitrogen (TN), ammonia nitrogen (NH_3_-N), and total phosphorus (TP) according to the Chinese national standard methods after filtration through a 0.45 μm membrane [30]. Residual ratio (%) of TN (or TP) was calculated after each measurement as (C/C_0_), where C is the concentration at time t and C_0_ is the concentration at time 0. Then, removal efficiency can be calculated as (1-(C/C_0_)).

All of the experiments were performed in triplicate. The data were displayed as the mean values with standard deviations. One-way analysis of variance (ANOVA) with LSD test was utilized to analyze the significant differences between the control group and different treatments. The statistically significant differences as compared to the control group were represented in the figures by * if *p* < 0.05 and ** if *p* < 0.01, respectively.

## 3. Results

### 3.1. The Removal of Total Nitrogen by S. platensis

**Figure 1:**
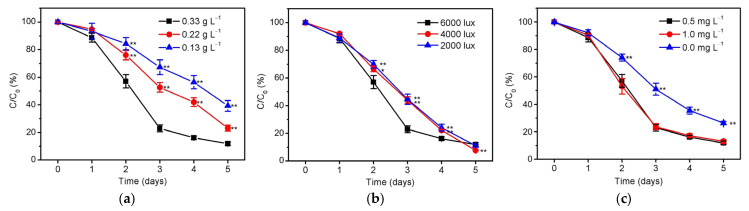
The residual ratio (C/C_0_) of total nitrogen in the culture solutions of *S. platensis* under different (**a**) initial inoculation amounts of 0.13–0.33 g/L (illumination intensity = 6000 lux, initial phosphorus level = 0.5 mg/L), (**b**) illumination intensities of 2000–6000 lux (initial inoculation amount = 0.33 g/L, initial phosphorus level = 0.5 mg/L), and (**c**) initial phosphorus levels of 0–1.0 mg/L (initial inoculation amount = 0.33 g/L, illumination intensity = 6000 lux) within 5 days cultivation. The statistically significant differences as compared to the optimized condition (initial inoculation amount = 0.33 g/L, illumination intensity = 6000 lux, and initial phosphorus level = 0.5 mg/L) were represented by * if *p* < 0.05 and ** if *p* < 0.01, respectively.

Reclaimed water is commonly obtained from the effluent of sewage treatment plant, which is at least subject to secondary treatment including nitrification [31]. Consequently, nitrate is almost the only form of total nitrogen in reclaimed water and is also reported to be the major contributor of total dissolved inorganic nitrogen in the aquatic environment [15,32]. Figure 1 shows the removal of *S. platensis* towards total nitrogen, whose initial amount is represented by 15 mg/L nitrate according to the national standard of China [17]. As can be seen in Figure 1a, the total nitrogen could be effectively removed by *S. platensis* after a one-day adaptation process. The removal efficiency increased by increasing the initial inoculation amount from 0.13 to 0.33 g/L algal biomass, resulting in the most significant difference (*p* < 0.01) at the 3rd day. Although the removal rate decreased slightly between the 3rd and 5th day, the initial inoculum amount as high as 0.33 g/L is still the best condition, and finally reached the 88.1 % removal efficiency of total nitrogen (about 1.8 mg/L left in the aqueous solution). This result can be easily understood since more microalgae inevitably need more nutrients such as nitrate to maintain their physiological activities, while the assimilation of nutrients should be also affected when they are limited [33,34]. The influence of illumination intensity was then studied as shown in Figure 1b, displaying the significant difference (*p* < 0.01) between 6000 lux and 4000/2000 lux at the 3rd day. The result suggested that the high illumination intensity could increase the removal efficiency of nitrate, which may be attributed to the penetration of the strong light for enhancing the light energy capture of microalgae until the saturation point [35]. It is worth mentioning that the phosphorus content in the reclaimed water is usually as low as 0.5–1.0 mg/L according to the national standard of China [17]. Therefore, the lack of phosphorus may cause the decline of nitrogen removal capability of *S. platensis* by taking into account the Stumm empirical formula (N/P mass ratio = 7.2) for microalgae [36]. Fortunately, *S. platensis* can effectively remove nitrate with 0.5–1.0 mg/L phosphorus regardless of the empirical formula, albeit the removal efficiency of total nitrogen has been significantly reduced (*p* < 0.01) without phosphorus (0.0 mg/L), as shown in Figure 1c.

### 3.2. The Removal of Total Phosphorus by S. platensis

**Figure 2:**
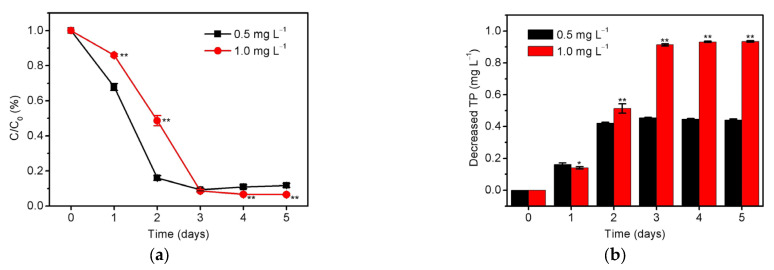
The (**a**) residual ratio (C/C_0_) and (**b**) decreased concentration of total phosphorus (initial phosphorus levels are 0.5 mg/L and 1.0 mg/L, respectively) in the culture medium of *S. platensis* within 5 days cultivation under initial inoculation amount of 0.33 g/L and illumination intensity of 6000 lux. The statistically significant differences as compared to the optimized condition (initial inoculation amount = 0.33 g/L, illumination intensity = 6000 lux, and initial phosphorus level = 0.5 mg/L) were represented by * if *p* < 0.05 and ** if *p* < 0.01, respectively.

To further study the effect of phosphorus content on the nutrient removal capability of *S. platensis*, the removal of total phosphorus was examined under the optimized conditions (0.33 g/L initial inoculation amount and 6000 lux illumination intensity), as can be seen in Figure 2a. Slightly different from the nitrogen removal, *S. platensis* seems to be more sensitive to phosphorus, displaying the more obvious removal of total phosphorus at the 1st day. By using the initial phosphorus content of 0.5 mg/L, the lowest limit of Grade IA national discharge standard of pollutants for municipal wastewater treatment plant of China [17], the removal efficiency increased rapidly to 84.0 % and 90.7 % at the 2nd and 3rd days, respectively. Additionally, the removal efficiency could also increase to 91.3 % by using the initial phosphorus content of 1.0 mg/L (upper limit of Grade IA national discharge standard). These results demonstrated that *S. platensis* could efficiently remove total phosphorus to less than 0.1 mg/L in three days. More importantly, *S. platensis* is found to be able to effectively remove nitrogen even when phosphorus is insufficient by combining the results of Figure 1 and Figure 2. For instance, *S. platensis* had a high nitrogen removal efficiency between the 2nd and 3rd days, when the total phosphorus content had decreased from 0.5 to 0.08 mg/L. However, it does not mean that *S. platensis* only requires little phosphorus to maintain growth and assimilate nitrogen. This can be clearly seen in Figure 2b that the decreased content of total phosphorus by *S. platensis* is significantly high (*p* < 0.01) when the initial phosphorus content is 1.0 mg/L. Hence, these results show that *S. platensis* has the strong adaptability for removing the excess nitrate in the case of phosphorus deficiency. This may be attributed to the fact that microalgae can maintain its basic physiological activity by using the endogenous phosphorus accumulated in the cell body [37,38]. From the above results, one can draw a conclusion that the phosphorus (0.5–1.0 mg/L) in the reclaimed water can be effectively controlled by *S. platensis*, and it is also an important factor to improve the removal efficiency of total nitrogen.

### 3.3. The Variation of Ammonia Content during the Cultivation Process

**Figure 3:**
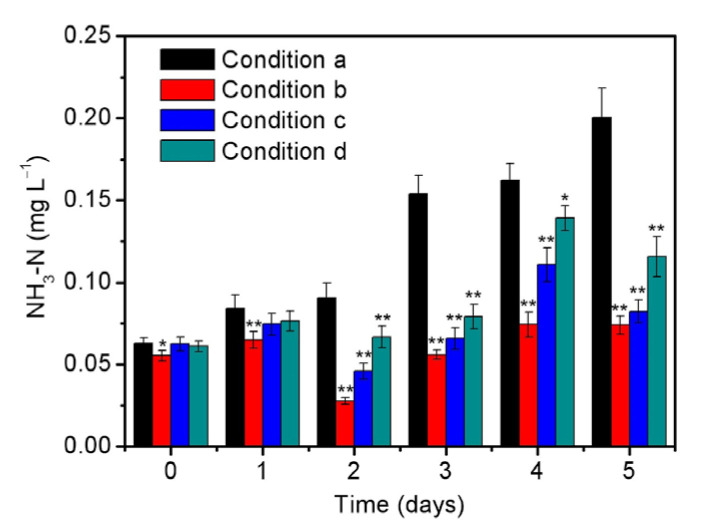
The contents of ammonia in the culture solutions of *S. platensis* within 5 days cultivation under the different conditions including (a) initial inoculation amount = 0.33 g/L, illumination intensity = 6000 lux, and initial phosphorus level = 0.5 mg/L; (b) initial inoculation amount = 0.13 g/L, illumination intensity = 6000 lux, and initial phosphorus level = 0.5 mg/L; (c) initial inoculation amount = 0.33 g/L, illumination intensity = 2000 lux, and initial phosphorus level = 0.5 mg/L; and (d) initial inoculation amount = 0.33 g/L, illumination intensity = 6000 lux, and initial phosphorus level = 0.0 mg/L. The statistically significant differences as compared to the optimized condition (condition a) were represented by * if *p* < 0.05 and ** if *p* < 0.01, respectively.

Previous studies have proposed that microalgae generally assimilated the nitrogen resource by converting ammonia into glutamine and nitrate needs to be reduced to ammonia by reductase before utilization [39]. Therefore, the ammonia nitrogen contents during the 5-day cultivation process were studied under different conditions as shown in Figure 3. Condition a is the optimal one (i.e., initial inoculation amount = 0.33 g/L, illumination intensity = 6000 lux, and initial phosphorus level = 0.5 mg/L). Condition b, c, and d changed initial inoculation amount, illumination intensity, and initial phosphorus level, respectively, compared with condition a. It is found that the initial level of ammonia nitrogen is approximately 0.06 mg/L, which may be attributed to the initially inoculated *S. platensis*. The content of ammonia nitrogen then increased to 0.2 mg/L under the optimized condition (0.33 g/L initial inoculation amount, 6000 lux illumination intensity, and 0.5 mg/L initial phosphorus level), confirming the gradual reduction in nitrate to produce ammonia that is beneficial to microalgae uptake [40]. In addition, negligible amount of nitrite could be detected in the culture solution, indicating the rate-limiting step should be the conversion of nitrate to nitrite instead of the further conversion to ammonia [41]. In comparison with the optimized condition, the content of ammonia nitrogen in Figure 3 also increased with time even using the lower initial inoculation amount, illumination intensity, and initial phosphorus level, resulting in the final amounts of ammonia at the 5th day that were much lower than 0.2 mg/L. All the above results suggested that *S. platensis* could rapidly remove total nitrogen without accumulating a large amount of ammonia, which would not exceed the standard limit (5 mg/L) and also not inhibit the growth of microalgae [17,42]. Consequently, *S. platensis* is considered to be a promising candidate to eliminate residual nutrients (e.g., nitrate and phosphorus) in reclaimed water without producing hazardous by-products (e.g., ammonia).

### 3.4. The Algal Growth under Different Conditions

**Figure 4:**
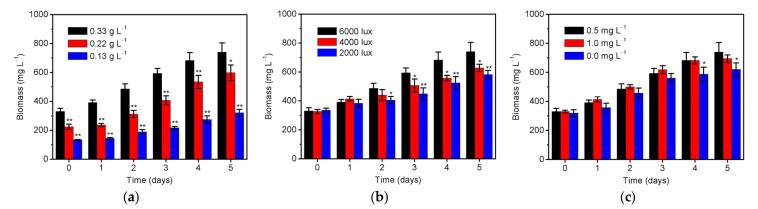
The biomass productivity of *S. platensis* under different (**a**) initial inoculation amounts of 0.13–0.33 g/L (illumination intensity = 6000 lux, initial phosphorus level = 0.5 mg/L), (**b**) illumination intensities of 2000–6000 lux (initial inoculation amount = 0.33 g/L, initial phosphorus level = 0.5 mg/L), and (**c**) initial phosphorus levels of 0–1.0 mg/L (initial inoculation amount = 0.33 g/L, illumination intensity = 6000 lux) within 5 days cultivation. The statistically significant differences as compared to the optimized condition (initial inoculation amount = 0.33 g/L, illumination intensity = 6000 lux, and initial phosphorus level = 0.5 mg/L) were represented by * if *p* < 0.05 and ** if *p* < 0.01, respectively.

The removal of nutrients by *S. platensis* can not only improve the quality of reclaimed water but also is a necessary process to maintain the algal growth, which in turn further promotes its remediation ability as shown in Figure 1. Therefore, the biomass productivity of *S. platensis* was investigated under different conditions as displayed in Figure 4. It is shown in Figure 4a that the biomass increased to 0.32, 0.60, and 0.74 g/L by using the initial inoculation amounts of 0.13, 0.22, and 0.33 g/L *S. platensis*, respectively. The result demonstrated that higher inoculation amount could lead to more biomass accumulation (Figure 4a) and further improve the removal efficiency of total nitrogen (Figure 1a). However, the massive amount of biomass inevitably aggravated the situation of nutrient deficiency [23]. It thus has to recognize that *S. platensis* seems to be always starvation when applying for removing nitrate from the reclaimed water. As the photoautotrophic micro-organism, illumination intensity is an important factor for influencing the biomass growth of *S. platensis*, and thus was also studied, as can be seen in Figure 4b. It is found that the microalgal biomass under high illumination intensity (6000 lux) started to be significantly higher (*p* < 0.05) than those under low illumination intensity (4000/2000 lux) from the 3rd day. This is in line with the expectation that the photosynthetic activity and specific growth rate commonly increased with the increment of illumination intensity before reaching the light saturation point [35]. The effect of initial phosphorus level on the growth of *S. platensis* was further examined as displayed in Figure 4c. The absence of phosphorus obviously caused a significant reduction (*p* < 0.05) in biomass from the 4th day, which is later than that in nitrogen removal (from the 2nd day in Figure 1c). The result showed that phosphorus has little effect on the growth of microalgae, which is also supported by comparable biomass growth and nitrogen removal no matter if 0.5 or 1.0 mg/L phosphorus was used [43].

### 3.5. The Variation of Photosynthesis Pigments

**Figure 5:**
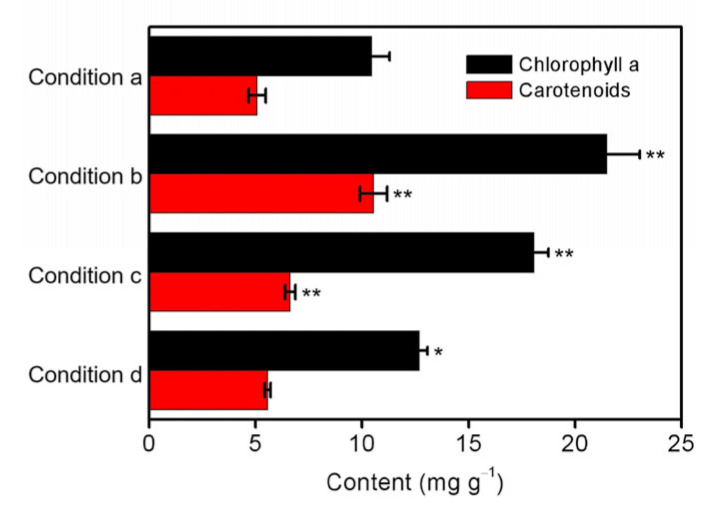
The contents of chlorophyll a and carotenoids in *S. platensis* after 5 days cultivation under the different conditions including (a) initial inoculation amount = 0.33 g/L, illumination intensity = 6000 lux, and initial phosphorus level = 0.5 mg/L; (b) initial inoculation amount = 0.13 g/L, illumination intensity = 6000 lux, and initial phosphorus level = 0.5 mg/L; (c) initial inoculation amount = 0.33 g/L, illumination intensity = 2000 lux, and initial phosphorus level = 0.5 mg/L; and (d) initial inoculation amount = 0.33 g/L, illumination intensity = 6000 lux, and initial phosphorus level = 0.0 mg/L. The statistically significant differences as compared to the optimized condition (condition a) were represented by * if *p* < 0.05 and ** if *p* < 0.01, respectively.

Photosynthesis is an important physiological process of algal growth and is the basis for microalgae to accumulate biomass [44]. The photosynthetic pigments including chlorophyll a and carotenoids have been recognized as the critical biomarkers for exogenous stress [45]. Consequently, the contents of these pigments in culture solutions were studied under different conditions. As shown in Figure 5, there are significant differences in comparison with the optimized condition by decreasing the initial inoculation amount to 0.13 g/L (*p* < 0.01), illumination intensity to 2000 lux (*p* < 0.01), and initial phosphorus level to 0.0 mg/L (*p* < 0.05). The result suggested that the phosphorus starvation stress could promote the photosynthetic reaction by increasing chlorophyll a content, which was also accumulated massively to cope with the low illumination intensity of 2000 lux to capture more light energy [46]. The high content of chlorophyll a under the initial inoculation amount of 0.13 g/L was then attributed to more nutrient supplement for less biomass of *S. platensis*, which coincided well with the result of carotenoids. In contrast to the findings obtained for chlorophyll a, the contents of carotenoids have almost no difference by adjusting the phosphorus level. It is well known that carotenoids are not only the photosynthetic pigments contributing to light harvesting with chlorophyll but also the antioxidants protecting the microalgal structure and function [47]. Therefore, the effect of phosphorus starvation stress on the growth of *S. platensis* and the removal efficiency of total nitrogen should be solely limited to photosynthetic efficiency rather than lipid peroxidation.

### 3.6. Effects of Background Ions on Biomass Growth and Nitrogen Removal

**Figure 6:**
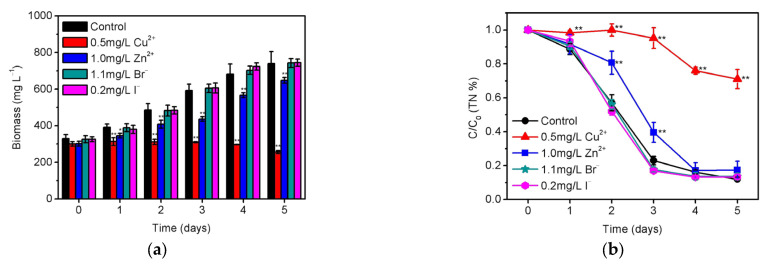
Effects of 0.5 mg/L Cu^2+^, 1.0 mg/L Zn^2+^, 1.1 mg/L Br^-^, and 0.2 mg/L I^-^ on the (**a**) biomass growth and (**b**) residual ratio (C/C_0_) of total nitrogen for *S. platensis* within 5 days cultivation under the optimized condition. The statistically significant differences as compared to the control group were represented by * if *p* < 0.05 and ** if *p* < 0.01, respectively.

Generally, the reclaimed water was obtained from sewage effluent, and thus should inevitably contain some background ions, which are likely to influence the physiological activity of *S. platensis* [48]. Hence, the effects of copper/zinc cations (Cu^2+^/Zn^2+^) and bromide/iodide anions (Br^-^/I^-^) on the biomass growth and total nitrogen removal were investigated as presented in Figure 6. The result illustrated that the biomass growth of *S. platensis* was significantly inhibited (*p* < 0.01) by 0.5 mg/L Cu^2+^ in Figure 6a, which is the limit of the national discharge standard of China [17]. This is why *S. platensis* did not remove total nitrogen in the first 3 days. Very interestingly, *S. platensis* displayed some removal ability of total nitrogen at the 5th day in Figure 6b, albeit the microalgal biomass has been significantly reduced compared to the initial inoculation amount. In comparison with Cu^2+^, *S. platensis* is found to be less sensitive to Zn^2+^ by considering the lower inhibition of 1.0 mg/L Zn^2+^ (standard limit of GB18918, 2002) towards biomass growth in Figure 6a. Moreover, *S. platensis* could effectively remove more than 90 % total nitrogen from the aqueous solution from the 3rd to 5th day after the initial 2-day adaptation in Figure 6b. The above results suggested the limited influence of 1.0 mg/L Zn^2+^ on *S. platensis* and its gradual recovery, while 0.5 mg/L Cu^2+^ should be definitely more toxic and would cause a more significant reduction in both biomass growth and nitrogen removal. This is consistent with the previous reports that microalgae including *Chlamydomonas*, *Scenedesmus*, and *Cladophora* were also more sensitive to Cu^2+^ than Zn^2+^; thus, 0.5 mg/L Cu^2+^ is already an unacceptable level for these aquatic micro-organisms [49,50,51]. In addition to the metal cations, the biomass growth of *S. platensis* was found to be hardly affected by 1.1 mg/L Br^-^ or 0.2 mg/L I^-^, both of which are almost the highest level reported in water and wastewater according to our previous survey [52]. Surprisingly, the significantly high removal efficiencies of total nitrogen were found by introducing these anions into the culture solutions. This may be due to the fact that *S. platensis* is a kind of halophilic micro-organism that is suitable for growing under high salinity [53]. It is thus proposed that more attention should be paid to the Cu^2+^ content and the corresponding impact when applying microalgae to improve the quality of reclaimed water.

## 4. Conclusions

In summary, we attempted to apply *S. platensis* in this work to remove low concentration of nitrate under nutrient-deficient conditions for further improving the quality of reclaimed water. The removal efficiency of 15 mg/L total nitrogen could be increased by increasing the initial inoculation amount to 0.33 g/L and illumination intensity to 6000 lux. The low level (0.5–1.0 mg/L) of phosphorus could be also controlled by *S. platensis* and be sufficient to help remove the nitrate. Under the optimized condition, *S. platensis* is able to effectively eliminate nitrate and phosphorus by producing extremely low hazardous by-product ammonia. More algal biomass was found to be accumulated by using higher inoculation amount (0.33 g/L) and illumination intensity (6000 lux), while 0.5–1.0 mg/L phosphorus has little effect on the biomass growth. The low illumination intensity of 2000 lux and phosphorus starvation stress were found to stimulate the photosynthetic system by increasing content of chlorophyll a but have almost no effect on lipid peroxidation by considering the stable content of carotenoids. The background cations of Zn^2+^ and particularly Cu^2+^ were found to inhibit the biomass growth and nitrate removal in reclaimed water, while the halophilic *S. platensis* is adaptable for growing in culture solutions with Br^-^/I^-^ anions. The current results are anticipated to become strong support by further employing *S. platensis* to improve the quality of reclaimed water.

## Data Availability

The data presented in this study are available upon request from the corresponding author.

## Supplementary Materials

The following supporting information can be downloaded at: https://www.mdpi.com/article/10.3390/ijerph20032117/s1, Table S1: The compositions of Zarrouk medium and synthetic reclaimed water used in this work and the corresponding Grade IA standard (GB18918-2002); Figure S1: Optical microscopy image of *S. platensis* at 100 × magnification; Figure S2: Schematic diagram of the experimental setup.

## References

[1] Grant S.B., Saphores J.D., Feldman D.L., Hamilton A.J., Fletcher T.D., Cook P.L.M., Stewardson M., Sanders B.F., Levin L.A., Ambrose R.F. (2012). Taking the “waste” out of “wastewater” for human water security and ecosystem sustainability. Science.

[2] Mekonnen M.M., Hoekstra A.Y. (2016). Four billion people facing severe water scarcity. Sci. Adv..

[3] van Loosdrecht M.C.M., Brdjanovic D. (2014). Anticipating the next century of wastewater treatment. Science.

[4] Nixdorff H., Noga J., Amsalu D., Springett J., Ashbolt N. (2021). Improving the implementation of water and resource recovery in Canada. J Water Reuse Desal..

[5] Wang X., Daigger G., Lee D.J., Liu J.X., Ren N.Q., Qu J.H., Liu G., Butler D. (2018). Evolving wastewater infrastructure paradigm to enhance harmony with nature. Sci. Adv..

[6] Liu L., Lopez E., Dueas-Osorio L., Stadler L., Xie Y.F., Alvarez P.J.J., Li Q.L. (2020). The importance of system configuration for distributed direct potable water reuse. Nat. Sustain..

[7] Sun D., Lin X., Lu Z., Huang J., Li G., Xu J. (2022). Process evaluation of urban river replenished with reclaimed water from a wastewater treatment plant based on the risk of algal bloom and comprehensive acute toxicity. J. Water Reuse Desal..

[8] Pisani P.L.D. (2006). Direct reclamation of potable water at windhoek’s Goreangab reclamation plant. Desalination.

[9] Leverenz H.L., Tchobanoglous G., Asano T. (2011). Direct potable reuse: A future imperative. J. Water Reuse Desal..

[10] Lavee D. (2010). A cost-benefit analysis of alternative wastewater treatment standards: A case study in Israel. Water Environ. J..

[11] Schnoor J.L. (2009). NEWater Future?. Environ. Sci. Technol..

[12] Lyu S.D., Chen W.P., Zhang W.L., Fan Y.P., Jiao W.T. (2016). Wastewater reclamation and reuse in China: Opportunities and challenges. J. Environ. Sci..

[13] Wang Z., Li J.S., Li Y.F. (2017). Using reclaimed water for agricultural and landscape irrigation in China: A review. Irrig. Drain..

[14] Conley D.J., Paerl H.W., Howarth R.W., Boesch D.F., Seitzinger S.P., Havens K.E., Lancelot C., Likens G.E. (2009). Controlling eutrophication: Nitrogen and phosphorus. Science.

[15] Jiang X.H., Song D.A., Dong M.Q., Li F.M. (2019). Feasibility analysis of further upgrading wastewater treatment plant executing the first level A standard: A case study of Qingdao. Environ. Eng..

[16] Wang J.H., Zhang T.Y., Dao G.H., Xu X.Q., Wang X.X., Hu H.Y. (2017). Microalgae-based advanced municipal wastewater treatment for reuse in water bodies. Appl. Microbiol. Biotechnol..

[17] (2002). The National Discharge Standard of Pollutants for Municipal Wastewater Treatment Plant.

[18] Beusen A.H.W., Bouwman A.F., Van Beek L.P.H., Mogollón J.M., Middelburg J.J. (2016). Global riverine N and P transport to ocean increased during the 20th century despite increased retention along the aquatic continuum. Biogeosciences.

[19] Li H.M., Tang H.J., Shi X.Y., Zhang C.S., Wang X.L. (2014). Increased nutrient loads from the Changjiang (Yangtze) River have led to increased harmful algal blooms. Harmful Algae.

[20] Gao G., Clare A.S., Rose C., Caldwell G.S. (2017). Eutrophication and warming-driven green tides (Ulva rigida) are predicted to increase under future climate change scenarios. Mar. Pollut. Bull..

[21] Vineyard D., Hicks A., Karthikeyan K.G., Barak P. (2020). Economic analysis of electrodialysis, denitrification, and anammox for nitrogen removal in municipal wastewater treatment. J. Clean Prod..

[22] Pennisi E. (2012). A better way to denitrify wastewater. Science.

[23] Wang Y.N., Pang H., Yu C., Wang J.H., Chi Z.Y., Xu Y.P., Li S.Y., Zhang Q., Che J. (2022). Growth and nutrients removal characteristics of attached Chlorella sp. using synthetic municipal secondary effluent with varied hydraulic retention times and biomass harvest intervals. Algal Res..

[24] Gao F., Yang Z.H., Li C., Zeng G.M., Ma D.H., Zhou L. (2015). A novel algal biofilm membrane photobioreactor for attached microalgae growth and nutrients removal from secondary effluent. Bioresource Technol..

[25] Klanchui A., Khannapho C., Phodee A., Cheevadhanarak S., Meechai A. (2012). i AK692: A genome-scale metabolic model of *Spirulina platensis C_1_*. BMC Syst. Biol..

[26] Jung F., Krüger-Genge A., Waldeck P., Kupper J.H. (2019). Spirulina platensis, a super food?. J. Cell. Biotechnol..

[27] Li X.T., Li W., Zhai J., Wei H.X., Wang Q.F. (2019). Effect of ammonium nitrogen on microalgal growth, biochemical composition and photosynthetic performance in mixotrophic cultivation. Bioresource Technol..

[28] Jiang X.H., Wang D.B., Wu W.R., Li F.M. (2022). Ecotoxicological effect of enrofloxacin on Spirulina platensis and the corresponding detoxification mechanism. Environ. Sci. Process. Impacts.

[29] Lightenthaler H.K. (1987). Chlorophylls and carotenoids: Pigments of photosynthetic biomembranes. Method. Enzymol..

[30] State Environmental Protection Administration (2002). Monitoring Method of Water and Wastewater.

[31] Mesquita D.P., Quintelas C., Amaral A.L., Ferreira E.C. (2017). Monitoring biological wastewater treatment processes: Recent advances in spectroscopy applications. Rev. Environ. Sci. Biotechnol..

[32] Teichberg M., Fox S.E., Olsen Y.S., Valiela I., Martinetto P., Iribarne O., Muto E.Y., Petti M.A.V., Corbisier T.N., Soto-Jimenez M. (2010). Eutrophication and macroalgal blooms in temperate and tropical coastal waters: Nutrient enrichment experiments with Ulva spp. Global Change Biol..

[33] Sun K.M., Li R.X., Li Y., Xin M., Xiao J., Wang Z.L., Tang X.X., Pang M. (2015). Responses of Ulva prolifera to short-term nutrient enrichment under light and dark conditions. Estuar. Coast. Shelf S..

[34] Fan H.H., Wang K., Wang C., Yu F.Y., He X.X., Ma J., Li X.T. (2020). A comparative study on growth characters and nutrients removal from wastewater by two microalgae under optimized light regimes. Environ. Technol. Innov..

[35] Olguín E.J., Galicia S., Angulo-Guerrero O., Hernández E. (2001). The effect of low light flux and nitrogen deficiency on the chemical composition of *Spirulina* sp. (*Arthrospira*) grown on digested pig waste. Bioresource Technol..

[36] Cai T., Park S.Y., Li Y. (2013). Nutrient recovery from wastewater streams by microalgae: Status and prospects. Renew. Sust. Energ. Rev..

[37] Oliver R.L., Ganf G.G., Whitton B.A., Potts M. (2002). Freshwater blooms. The Ecology of Cyanobacteria: Their Diversity in Time and Space.

[38] Wu Y.H., Yu Y., Li X., Hu H.Y., Su Z.F. (2012). Biomass production of a Scenedesmus sp. under phosphorous-starvation cultivation condition. Bioresource Technol..

[39] Lu Q., Chen P., Addy M., Zhang R.C., Deng X.Y., Ma Y.W., Cheng Y.L., Hussain F., Chen C., Liu Y.H. (2018). Carbon-dependent alleviation of ammonia toxicity for algae cultivation and associated mechanisms exploration. Bioresource Technol..

[40] Bloom A.J., Sukrapanna S.S., Warner R.L. (1992). Root respiration associated with ammonium and nitrate absorption and assimilation by barley. Plant Physiol..

[41] Bagchi S.N. (1994). Cyanobacterial nitrate reduction: Process and regulation. Proc. Ind. Sci. Acad..

[42] Belkin S., Boussiba S. (1991). Resistance of *Spirulina platensis* to ammonia at high pH values. Plant Cell Physiol..

[43] Li R., Wu X., Wei Q., Wang Z., Li Y., Sun P. (2009). Growth of *Enteromorpha prolifera* under different nutrient conditions. Adv. Mar. Sci..

[44] Djaghoubi A., Bouhoun M.D., Said S.H., Saggaï A., Sobti S., Aissa B.H. (2015). Growth and Nitrogen Removal Efficiency as Protein Content of Spirulina from Tertiary Municipal Wastewater in Ouargla (Algerian Bas-Sahara). Energy Procedia.

[45] Shashirekha V., Sridharan M.R., Swamy M. (2015). Biochemical response of cyanobacterial species to trivalent chromium stress. Algal Res..

[46] Ferreira V.S., Pinto R.F., Sant’Anna C. (2016). Low light intensity and nitrogen starvation modulate the chlorophyll content of Scenedesmus dimorphus. J. Appl. Microbiol..

[47] Xiong J.Q., Kurade M.B., Jeon B.H. (2017). Ecotoxicological effects of enrofloxacin and its removal by monoculture of microalgal species and their consortium. Environ. Pollut..

[48] El-Agawany N.I., Kaamoush M.I.A., El Salhin H. (2022). Impact of heavy metal pollution on growth, biochemical composition and nutrition quality of *Spirulina platensis*. https://assets.researchsquare.com/files/rs-1737846/v1/c51e2272-40fe-4c16-8f93-0062f555fc95.pdf?c=1658375969.

[49] Danilov R.A., Ekelund N.G.A. (2001). Effects of Cu^2+^, Ni^2+^, Pb^2+^, Zn^2+^ and pentachlorophenol on photosynthesis and motility in Chlamydomonas reinhardtii in short-term exposure experiments. BMC Ecol..

[50] Tripathi B.N., Mehta S.K., Gaur J.P. (2004). Recovery of uptake and assimilation of nitrate in *Scenedesmus* sp. previously exposed to elevated levels of Cu^2+^ and Zn^2+^. J Plant. Physiol..

[51] Cao D.J., Xie P.P., Deng J.W., Zhang H.M., Ma R.X., Liu C., Liu R.J., Liang Y.G., Li H., Shi X.D. (2015). Effects of Cu^2+^ and Zn^2+^ on growth and physiological characteristics of green algae, Cladophora. Environ. Sci. Pollut. R..

[52] Jiang X.H., Song D.A., Wang D.B., Zhang R.M., Fang Q.N., Sun H.Q., Kong F.Y. (2020). Eliminating imidacloprid and its toxicity by permanganate via highly selective partial oxidation. Ecotox. Environ. Saf..

[53] Coppens J., Lindeboom R., Muys M., Coessens W., Alloul A., Meerbergen K., Lievens B., Clauwaert P., Boon N., Vlaeminck S.E. (2016). Nitrification and microalgae cultivation for two-stage biological nutrient valorization from source separated urine. Bioresource Technol..

