# Supplementary material for: Improving the Quality of Reclaimed Water via Applying Spirulina platensis to Eliminate Residual Nitrate
Source: Int J Environ Res Public Health. 2023 Jan 24;20(3):2117. doi: 10.3390/ijerph20032117 (PMC9916132; doi:10.3390/ijerph20032117)
Supplement: Supplementary file 1 [file ijerph-20-02117-s001.zip › ijerph-2144584-supplementary.pdf]

Improving the quality of reclaimed water via applying *Spirulina platensis* to eliminate residual nitrate

Xiaohua Jiang[1], Xin Shan[1], Fengmin Li[1,2,]*

1 College of Environmental Science and Engineering, Ocean University of China, Qingdao 266100, China

2 Sanya Oceanographic Institution, Ocean University of China, Sanya 572000, China

* Corresponding author: lifengmin@ouc.edu.cn

Table S1. The compositions of Zarrouk medium and synthetic reclaimed water used in this work and the corresponding Grade IA standard (GB18918-2002).

	Zarrouk medium	Synthetic reclaimed water	Grade IA standard (GB18918-2002)
$NaHCO_3$	13.61 g/L	13.61 g/L	N.S.
Na_2CO_3	4.03 g/L	4.03 g/L	N.S.
K_2HPO_4	0.5 g/L	=0.5-1.0mg/L P	0.5-1.0mg/L P
$NaNO_3$	2.5 g/L	=15mg/L N	15mg/L N
K_2SO_4	1.0 g/L	1.0 g/L	N.S.
NaCl	1.0 g/L	1.0 g/L	N.S.
$MgSO_4 \cdot 7H_2O$	0.2 g/L	0.2 g/L	N.S.
$CaCl_2 \cdot 2H_2O$	0.04 g/L	0.04 g/L	N.S.
$FeSO_4 \cdot 7H_2O$	0.01 g/L	0.01 g/L	N.S.
H_3BO_3	2.86 mg/L	2.86 mg/L	N.S.
$MnCl_2 \cdot 4H_2O$	1.86 mg/L	=0.51 mg/L Mn	2.0 mg/L Mn
$ZnSO_4 \cdot 7H_2O$	0.22 mg/L	=0.05 mg/L Zn	1.0 mg/L Zn
$Na_2MoO_4 \cdot 2H_2O$	0.39 mg/L	0.39 mg/L	N.S.
$CuSO_4 \cdot 5H_2O$	0.08 mg/L	=0.02 mg/L Cu	0.5 mg/L Cu
$Co(NO_3)_2 \cdot 6H_2O$	0.05 mg/L	0.05 mg/L	N.S.

N. S. represents not specified in the Grade IA standard (GB18918-2002).

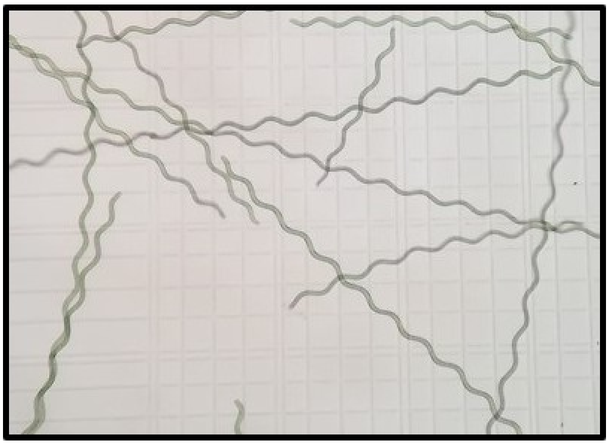

Figure S1. Optical microscopy image of *S. platensis* at 100 × magnification.

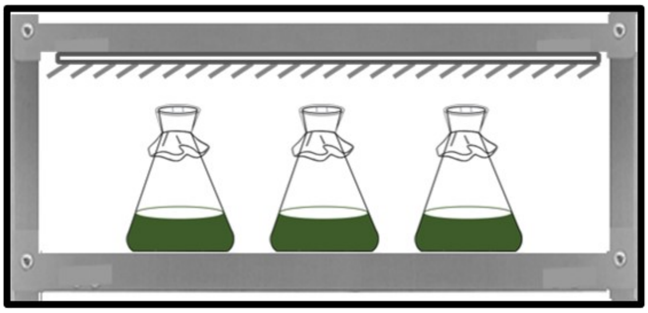

Figure S2. Schematic diagram of the experimental setup (Algae are cultured in conical flasks, which are placed on shelves in a small room. The temperature is controlled by an air conditioner, and the light is controlled by the light tube on each shelf. To ensure the light intensity and control the interaction between different treatment groups, each shelf is covered with a light curtain).
